# Detecting rare carnivores using scats: Implications for monitoring a fox incursion into Tasmania

**DOI:** 10.1002/ece3.3694

**Published:** 2017-12-05

**Authors:** David S. L. Ramsey, Candida Barclay, Catriona D. Campbell, Elise Dewar, Anna J. MacDonald, Elodie Modave, Sumaiya Quasim, Stephen D. Sarre

**Affiliations:** ^1^ Department of Environment, Land, Water and Planning Arthur Rylah Institute Heidelberg VIC Australia; ^2^ School of Biological Sciences University of Adelaide Adelaide SA Australia; ^3^ Department of Primary Industries, Parks, Water and Environment Invasive Species Branch Prospect TAS Australia; ^4^ Institute for Applied Ecology University of Canberra Canberra ACT 2617 Australia

**Keywords:** Bayesian analysis, detection probability, predator feces, scats, survival analysis, trace DNA, *Vulpes vulpes*

## Abstract

The ability to detect the incursion of an invasive species or destroy the last individuals during an eradication program are some of the most difficult aspects of invasive species management. The presence of foxes in Tasmania is a contentious issue with recent structured monitoring efforts, involving collection of carnivore scats and testing for fox DNA, failing to detect any evidence of foxes. Understanding the likelihood that monitoring efforts would detect fox presence, given at least one is present, is therefore critical for understanding the role of scat monitoring for informing the response to an incursion. We undertook trials to estimate the probability of fox scat detection through monitoring by scat‐detector dogs and person searches and used this information to critically evaluate the power of scat monitoring efforts for detecting foxes in the Tasmanian landscape. The probability of detecting a single scat present in a 1‐km^2^ survey unit was highest for scat‐detector dogs searches (0.053) compared with person searches ($\overset{¯}{x}≅0.015$) for each 10 km of search effort. Simulation of the power of recent scat monitoring efforts undertaken in Tasmania from 2011 to 2015 suggested that single foxes would have to be present in at least 20 different locations or fox breeding groups present in at least six different locations, in order to be detected with a high level of confidence (>0.80). We have shown that highly structured detection trials can provide managers with the quantitative tools needed to make judgments about the power of large‐scale scat monitoring programs. Results suggest that a fox population, if present in Tasmania, could remain undetected by a large‐scale, structured scat monitoring program. Therefore, it is likely that other forms of surveillance, in conjunction with scat monitoring, will be necessary to demonstrate that foxes are absent from Tasmania with high confidence.

## 1 INTRODUCTION

The ability to detect the incursion of an invasive species or destroy the last few individuals during an eradication program are some of the most difficult aspects of invasive species management (Cruz, Carrion, Campbell, Lavoie, & Donlan, 2009; Morrison, MacDonald, Walker, Lozier, & Shaw, 2007; Ramsey, Parkes, & Morrison, 2009). Hence, the ability to assess the effectiveness of various monitoring tools in areas where the target species occurs at low density is vitally important, as this guides the selection of appropriate tools to target those individuals and achieve effective management (Parkes et al., 2010). Recently, evidence of an incursion of the European red fox (*Vulpes vulpes*) into Tasmania (Sarre, MacDonald, Barclay, Saunders, & Ramsey, 2013; Saunders, Lane, Harris, & Dickman, 2006) led to the establishment of a specific program charged with the task of effecting eradication (Brown, Ramsey, & Gaffney, 2014). As part of this program, predator scat surveys were used extensively to infer the distribution of foxes (Sarre et al., 2013). Fox scats are morphologically similar to other extant predators in Tasmania (e.g., feral cats, *Felis catus*; quolls, *Dasyurus spp*, and Tasmanian devils, *Sarcophilus harrisii*), and hence, scats are subject to a mitochondrial DNA‐based test for species identification (Berry, Sarre, Farrington, & Aitken, 2007; Sarre et al., 2013). From *c*.13,000 predator scats submitted for testing, 61 were positively identified as “fox” by the DNA test. As a result of the widespread occurrence of physical evidence of foxes (primarily scats and four fox carcasses), a broad‐scale fox baiting program was implemented in 2010 using buried 1080 baits (Marks, Edwards, Obendorf, Pereira, & Hall, 2014; Parkes & Anderson, 2009). In mid‐2013, broad‐scale baiting ceased in Tasmania and efforts were shifted to landscape‐scale scat monitoring to confirm the absence of foxes (DPIPWE 2014). Presently, there has been no new evidence of foxes found since July 2011, despite extensive scat monitoring search effort undertaken since then.

Scat monitoring in Tasmania relies primarily on scat‐detector dogs and/or human observers searching an area for fox scats. Most searches conducted have fallen into two classes: structured surveys using a systematically placed grid of 3 × 3 km cells covering larger areas (landscape‐scale monitoring) or unstructured surveys in response to reported fox sightings (investigations) (Brown et al., 2014; Sarre et al., 2013). The last confirmed fox‐positive scat was found in July 2011 and since then, structured monitoring has been conducted in over 1000, 3 × 3 km monitoring units covering the majority of putative fox habitat in Tasmania, with no evidence of foxes found to date. This raises the question of the likelihood of detecting a fox using landscape‐scale scat monitoring, should one be present in Tasmania. This question has value not only in assessing the power of recent monitoring efforts but is also useful for the design of monitoring programs to detect future incursions (Field, Tyre, & Possingham, 2005; Wintle, Walshe, Parris, & Mccarthy, 2012). In addition, estimates of the detectability of scat monitoring are essential if we wish to make inferences about the probability of fox absence, if scat monitoring does not detect any individuals (Ramsey, Parkes, Will, Hanson, & Campbell, 2011; Ramsey et al., 2009; Regan, McCarthy, Baxter, Dane Panetta, & Possingham, 2006; Rout, Kirkwood, Sutherland, Murphy, & McCarthy, 2014; Rout, Moore, & McCarthy, 2014). Ideally, inferences about fox absence should also include a variety of other sources of monitoring data to make robust inference (Caley & Barry, 2014; Caley, Ramsey, & Barry, 2015).

Estimating detectability from monitoring data can be undertaken using a variety of field methods, usually involving repeat surveys with fixed effort and/or collection of ancillary data on individuals (Buckland, Anderson, Burnham, & Laake, 1993; MacKenzie et al., 2006; Otis, Burnham, White, & Anderson, 1978; Royle, 2004). Alternatively, studies of static targets such as plants and animal sign usually examine how the detection rate changes with increasing search effort (Garrard, Bekessy, McCarthy, & Wintle, 2008; Guillera‐Arroita, Ridout, Morgan, & Linkie, 2012; Moore, Hauser, Bear, Williams, & McCarthy, 2011; Regan et al., 2006). In the latter situation, detection experiments can often be useful for elucidating the relationship between detection probability and search effort (Garrard et al., 2008; Moore et al., 2011). In previous studies, scat detection experiments have been conducted under controlled conditions where scats are placed on predefined transects that were subject to searching. This ensured that each scat was encompassed by a relatively small potential search area (Long, Donovan, Mackay, Zielinski, & Buzas, 2007; Reed, Bidlack, Hurt, & Getz, 2011; Vynne et al., 2011). However, to our knowledge, scat detection rates have not been estimated under actual operational conditions, where the potential search areas could be large, as would be expected under large‐scale monitoring programs.

Here, we conduct an experimental assessment of the relationship between scat detection probabilities and search effort by scat‐detector dogs and person searches evaluated during landscape‐scale scat monitoring in Tasmania. We then use these estimates to assess the likelihood that scat monitoring search effort expended since 2011 in Tasmania would have detected foxes, if they were present, and use this to make recommendations for future scat monitoring programs.

## 2 MATERIALS AND METHODS

### 2.1 Structured and unstructured scat monitoring programs

In response to evidence of fox presence, the Tasmanian government undertook landscape‐scale monitoring of “priority” fox habitat in eastern and northern Tasmania to determine the spatial extent of the incursion (structured monitoring) (DPIPWE 2014). Priority habitat (optimal fox habitat based on expert opinion (Saunders et al., 2006) was subdivided into 3 × 3 km survey units and a subset of these were selected for structured surveys (with a random start point). These surveys were conducted across two time periods: Surveys 1: 2008–2010 (reported in Sarre et al., 2013) and Surveys 2: 2011–2015. The latter surveys repeated a subset of units from the first surveys but also extended the area of Tasmania covered. In addition, unstructured monitoring was undertaken in response to reports of fox sightings from the public. All fox sighting reports were subject to some degree of investigation. Since July 2011, there have been 757 recorded reports of fox sightings by the public. Sighting reports were investigated using a variety of methods, but only the 237 reports investigated using scat searching were considered here. These investigations were undertaken at a smaller scale than the structured surveys above, usually on a 1 × 1 km survey unit centerd on the location of the reported sighting.

Scat detection within survey units (either 3 × 3 or 1 × 1 km units) was undertaken by two‐person search teams or by trained scat‐detector dogs. Search effort differed between the search team types and the survey unit sizes. For person teams: ten person hours for 3 × 3 km units, and five person hours for 1 × 1 km units; for dog teams: 10–12 km for 3 × 3 km units, and five to six km for 1 × 1 km units. Monitoring within a unit (whether by dog or person teams) targeted “linear” features in the habitat (e.g., fence‐lines, water bodies, tracks, and hedgerows) as foxes tend to deposit more scats within or near these features (Webbon, Baker, & Harris, 2004). The survey starting point was haphazard, based on logistics, while search trajectories were generally preplanned based on aerial photography of the survey unit, with the aim of covering an array of the best “habitat features” available, while ensuring a reasonable coverage of the unit. For each survey unit monitored, search teams recorded the actual search path traversed using hand‐held GPS units (track logs). All carnivore scats encountered by person teams were collected, dried, and subjected to DNA testing (Berry et al., 2007; Sarre et al., 2013) whereas for dog search teams, only scats for which dogs produced a “sit” response (see below) were collected and subjected to DNA analysis. The DNA analysis method applied to scats collected during person surveys in 2014 differed from that used previously, in that scats were swabbed at the point of collection. DNA was extracted from each swab and subjected to DNA testing. This swabbing approach has been shown to be more rapid than DNA extraction directly from scats, without a loss of detectability (Ramón‐Laca, Soriano, Gleeson, & Godoy, 2015).

### 2.2 Dog training

Six Labrador dogs were involved at different times in fox scat detection work in Tasmania. Labrador dogs are ideal for this type of scent detection work because they are a robust breed with very good scenting ability and a naturally high play and food drive (Dahlgren et al., 2012; Mathews et al., 2013). Individual dogs were selected as puppies and gradually introduced to the single target of fox odor from approximately three months of age. Scent training was achieved by the association of the target odor paired with a food and/or play reward. Aversion methods were used to train dogs to ignore wildlife, snakes, and food when working in the field. Dogs were trained to indicate a target with a “sit response.” They were also trained to pinpoint the target by placing their nose on the ground next to the target scat with the command “show me.”

Two dogs were paired with a single handler, who was able to read their behavior and guide them through monitoring operations. Dogs and handlers were tested for proficiency every 3 months and formally “validated” by an outside qualified auditor every 12 months to ensure that they were operating at a satisfactory level.

### 2.3 Fox scat detection trials

Trials to estimate the probability of scat detection by scat‐detector dogs or two‐person search teams were undertaken for survey units at both 1 × 1 and 3 × 3 km scales. Trials were initially undertaken at four dedicated 1 × 1 km trial sites containing a relatively high numbers of trial scats (nine to 15 for dog teams and 30 scats for people teams—Table 1). For these trials, searchers were aware that they were being tested. Subsequently, trials were undertaken as part of the structured monitoring program to obtain a more realistic estimate of detection rates. These trials were undertaken by randomly selecting units from the pool designated to be monitored in a given week. Searchers were unaware on any given day whether a survey unit would contain trial “fox” scats.

**Table 1:** The number of monitoring trials and trial scats used and mean search distances for the scat detection trials for each monitoring unit size and team type

Unit size	Team type	No. of trials	No. of trial units	Total scats	Mean and (range) of scats per trial unit	Mean search distance (km)
1 km	Dog	60	42	363	6.1, (1–15)	6.0
1 km	Person	16	10	259	16.2, (1–30)	23.9
3 km	Dog	45	45	145	3.2, (1–7)	11.3
3 km	Person	21	21	104	4.9, (1–9)	17.9

Each trial involved a “controller” secretly placing a number of “fox” scats on randomly selected linear features within the designated survey unit (Table 1). Actual fox scats were used for trials involving scat detection dogs; these were brought into Tasmania periodically (under special authority according to section 19 of the Animal Health Act 1995) and stored in a secure facility. Nonfox carnivore scats (such as dog, cat, or quoll) were used for person trials. Upon placing a “fox” scat in the environment, the controller recorded the GPS coordinates of the location and took close‐up photographs of the scat in situ. Searchers, either a “person team” (two people) or a “dog team” (scat‐detector dog and handler) arrived independently at the survey unit and chose a selection of features within the unit to search as part of the survey. Searchers recorded GPS locations of all “fox” scats encountered as well as the route taken during the search (GPS track log) allowing for calculation of the cumulative distance searched up to that point. The GPS coordinates and photos of scats found (and collected) were compared to those taken by the controller when placing the scats in the field allowing the number of trial scats found by each search team to be determined. Every effort was made to recover any fox scat placed in the field that was not otherwise found. This was not always possible for a variety of reasons including scat destruction by insect activity, local disturbance by stock, or heavy rain.

### 2.4 Analysis

The cumulative distance searched to find each of the trial scats was treated as the “failure time” and used in a survival analysis. The analogy was that each scat was considered to be “alive” at the start of searching and that detection of the scat by the searchers resulted in the “death” of the scat, with the distance searched to that point treated as the survival time. Search distance was used rather than search time, because detection probability was likely to be directly related to area searched. However, as searchers were constantly moving, search distance and search time were likely interchangeable. As searchers started at random locations with respect to scat locations and searching occurred at a relatively constant rate, the distribution of survival distances $S\left(d\right)$ would be expected to follow an exponential distribution.

(1) $$S\left(d\right)=\exp\left(-\lambda d\right),$$

where λ is the detection rate (i.e., to the first detection) and *d* is distance. This model assumes that the detection rate is constant over all distances searched. However, if searching efficiency decreases with distance searched (e.g., due to fatigue), then the detection rate may decrease with distance, which can be accommodated by adopting a Weibull distribution for the survival distances.

(2) $$S\left(d\right)=\exp\left(-\lambda d^{\alpha }\right)$$

where the shape parameter α allows the detection rate to decrease (or increase) with distance searched. All scats not detected by searchers were treated as right‐censored observations with a censored survival distance equal to the maximum distance searched in the unit by that team. For these models, the scat detection rate λ was made to be a function of team type (dog or person teams) and grid size (3 km vs. 1 km), using a log link. As multiple scats were placed in each survey unit (Table 1), the detection rate was dependent on the number of scats placed at each unit (i.e., more scats would lead to a shorter distance searched before detecting the first scat). Assuming *n* scats are placed in each survey unit and scats are detected independently, then the detection distances within survey units can be modeled by substituting λ with *n*λ in Equations (1) and (2) (e.g., McCarthy et al., 2013). In addition, we also treated data for each trial within a survey unit as a random effect (some units were subject to multiple trials). The random effects for trial served the purpose of capturing residual variation due to differences in vegetation, features, terrain, and weather among trials.

Survival models were fitted to the data using Markov chain Monte Carlo methods in JAGS version 4.2.0 (Plummer, 2003). Weakly informative normal priors *N*(0, 10) were placed on fixed regression coefficients with a gamma prior *Ga*(1, 0.01) used for the Weibull shape parameter α. The trial ID random effects were assumed to be normally distributed with mean zero and standard deviation σ, with an uniform prior distribution *U*(0, 20) used for this parameter. Three MCMC chains were run and checked for convergence using the Brook—Gelman—Rubin convergence diagnostic (Brooks & Gelman, 1998) with convergence achieved after 20,000 iterations (<1.01 for all parameters). Thereafter, sampling continued for a further 10,000 iterations giving 30,000 samples for posterior summaries. JAGS code for the exponential and Weibull survival models are provided in the supplementary materials (Appendix S1). We compared the relative fit of the exponential and Weibull models using the widely applicable information criterion (WAIC) calculated using the R package loo (Vehtari, Gelman, & Gabry, 2016). WAIC is an improvement on the deviance information criterion (DIC) that is popularly used for Bayesian model comparison (e.g., Spiegelhalter, Best, Carlin, & Van Der Linde, 2002). Unlike DIC, which is based on the log likelihood for a point estimate of the parameters, WAIC is fully Bayesian, being based on the log likelihood evaluated across the posterior distribution of the parameter values. Asymptotically, WAIC is equivalent to Bayesian leave‐one‐out cross‐validation and hence, is a better reflection of the relative predictive performance of a model than is DIC (Vehtari et al., 2016). We calculated the difference in the expected predictive accuracy of the two models by calculating the difference in WAIC and associated standard error (Vehtari et al., 2016). The goodness of fit of the most supported model was calculated using posterior predictive distributions. This involved drawing replicated data from the generating model *y* ^rep^, the same size as the original data *y* and comparing the discrepancy between the observed and replicated data with a chi‐squared discrepancy measure and calculating a Bayesian *p* value (Gelman, Meng, & Stern, 1996).

### 2.5 Power of scat monitoring strategies to detect foxes

Although the scat detection trials described above estimate the probability that monitoring will detect a fox scat, this is not the same as estimating the probability that monitoring will detect a fox, given one is present. The probability of detecting one or more foxes using scat detection surveys will also depend on the following:

1. The minimum number of foxes that could reasonably constitute an extant fox population. We used values that encompass a single fox to a social group (see *Parameters* below).
2. The number of survey units where scats are likely to be deposited by a resident fox or fox group. This will depend on both the survey unit size and home range size.
3. The expected number of scats that are available for detection within a survey unit that is occupied by a fox. We used the estimate derived recently under Tasmanian conditions by Brown et al. (2014) in which the expected number of fox scats within a fox home range was estimated as the equilibrium between the scat deposition rate by a fox and the scat degradation rate in the environment (see Appendix S3).
4. The sensitivity of the mtDNA diagnostic test used to assign a putative predator scat as belonging to a fox (i.e., the probability that a fox scat is successfully diagnosed as a fox). This was estimated recently by Ramsey, MacDonald, Quasim, Barclay, and Sarre (2015) to be 0.85.

Points 1–4 above constitute the information necessary for defining both the number of fox scats that are at risk of being detected through scat monitoring and their spatial distribution in the environment.

**Figure 1:**
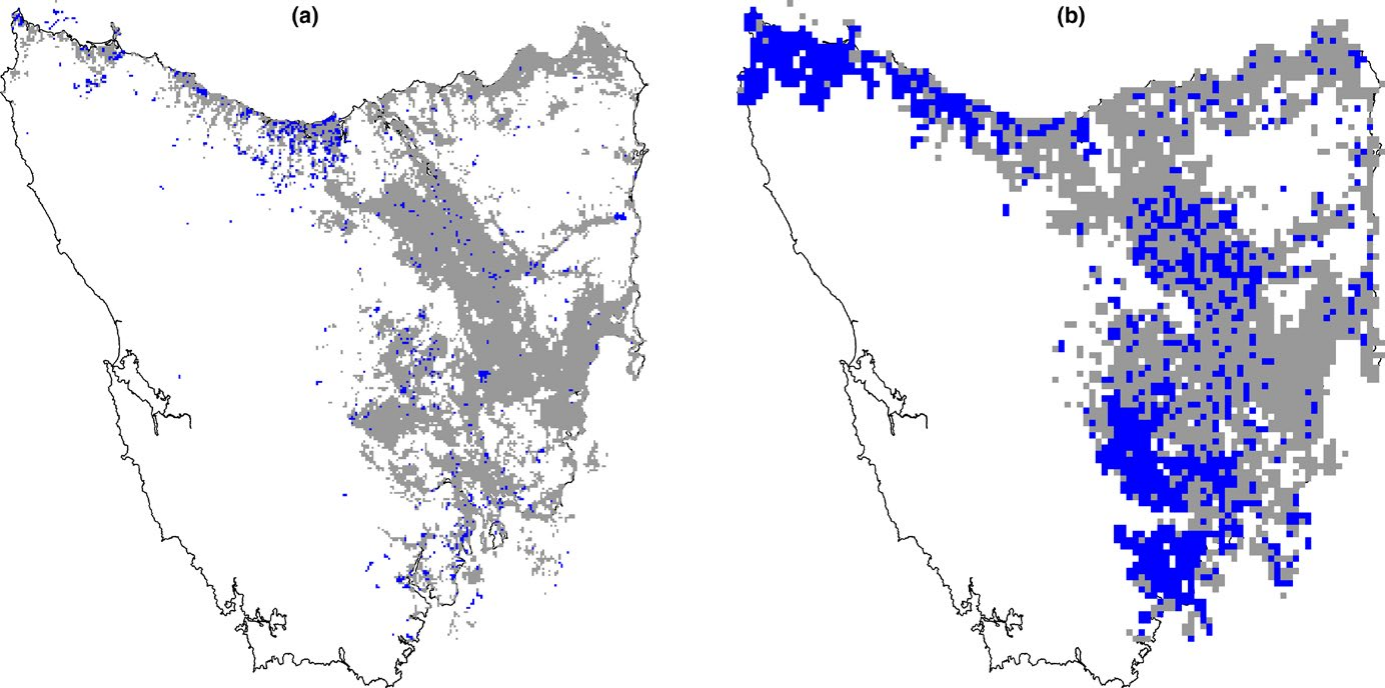
(a) Priority fox habitat estimated for Tasmania delineated in (a) 1 × 1 km and (b) 3 × 3 km survey units (gray shading). Blue shaded cells indicate monitoring units subject to scat detection surveys between 2011 and 2015

We used Monte Carlo simulation techniques to evaluate the likelihood that structured and unstructured scat monitoring conducted between 2011 and 2015 would detect a fox(s) or fox family group(s), given they were present within the region of priority fox habitat. This was possible as all teams undertaking both structured and unstructured scat monitoring recorded actual search paths (track logs) during monitoring activities. Track log data were overlaid on the map of priority fox habitat delineated into 1 × 1 km units, and the total distance searched for each unit that intersected a track log was calculated (Figure 1a). This was then repeated for the 3 × 3 km map of priority fox habitat (Figure 1b). Track logs often intersected more than one survey unit as tracks often meandered into survey units adjacent to the unit that was the focus of monitoring. We included all survey units that had at least some monitoring effort, even if only a small amount, in the power analysis. The design of the simulation study then used the following algorithm

1. Randomly place a fox(s) or fox group(s) home range (a circle of given radius for convenience) within the area of priority fox habitat divided into all potential 1 × 1 km monitoring units (i.e., Figure 1a).
2. Determine how many survey units were intersected by the fox home range.
3. Place a random number of fox scats, based on the expected number to be extant in the environment (net between production and degradation), in units overlapped by the fox home range according to distribution type (see below).
4. Place the sampling design over the landscape (i.e., actual sampled 1 × 1 or 3 × 3 km units). For sampled 3 × 3 km units, the number of fox scats “at risk” of detection was calculated by aggregating the scats from the nine 1 × 1 km units that corresponded to the location of the sampled 3 × 3 km unit.
5. If a sampled survey unit coincides with a unit intersected by a fox home range, then scats in the unit are detected with probability estimated from the scat detection trials undertaken above, given the actual distance searched.
6. Each detected “fox scat” was then subject to a simulated mtDNA test which assigns the scat as belonging to a fox with a designated probability (test sensitivity).
7. Replicate the simulated monitoring design multiple times, drawing random samples from distributions of model parameters at each iteration to incorporate their uncertainty in the estimated probabilities of fox detection.

### 2.6 Parameters

We varied several parameters to determine their effect on the power of recent scat monitoring efforts to detect fox presence, including the following:

1. Number of single foxes or fox groups (home ranges) (between 1 and 20)
2. Home range size of single foxes (120, 470, 1000, 1900 ha)
3. Distribution of scats deposited within the home range by a fox (random, clumped)

For the purposes of this study a fox “group” was defined as a family group consisting of an adult male/female pair and 4 cubs. The number of cubs is consistent with the mean litter size for adult females in Australia (Saunders, Kinnear, Braysher, & Coman, 1995). The home range size of a family group was also defined as 1.5 times the size of a single fox range size, varied as per (2) above. For each combination of these parameters, 10,000 replicated scat detection surveys were simulated, with each simulated survey varying the locations of foxes, the expected number of fox scats per home range (varying scat deposition and degradation), and the scat detection probability, given actual search distances (estimated from the detection trials above), which were drawn from predefined probability distributions. All simulations were conducted using R version 3.2.2 (R Development Core Team 2015). More details regarding the Monte Carlo algorithm, parameters, and distributions used in the simulation study are provided in the supplementary materials (Appendix S2). Finally, we also investigated how sensitive the estimated detection probability from the simulation study was to variation in the key parameters of the Monte Carlo algorithm using sensitivity analysis. We performed a global sensitivity analysis using the fourier amplitude sensitivity test (FAST) (Saltelli, Tarantola, & Chan, 1999) using the R package fast (Saltelli, Chan, & Scott, 2000). Further details of the sensitivity analysis are given in the supplementary materials (Appendix S4).

## 3 RESULTS

### 3.1 Fox scat detection trials

A total of 142 scat detection trials were undertaken using a total of 871 “fox” scats. Four different dog teams undertook 105 detection trials across 87 survey units, searching for an average of 6.0 and 11.3 km on 1‐km and 3‐km survey units, respectively. Twenty different person search teams undertook 37 detection trials across 31 survey units, searching for an average of 23.9 and 17.4 km for 1‐km and 3‐km survey units, respectively (Table 1). The longer average searching distance for person teams on 1‐km units was a reflection of higher average search distances undertaken at the four dedicated 1‐km detection trial sites.

**Figure 2:**
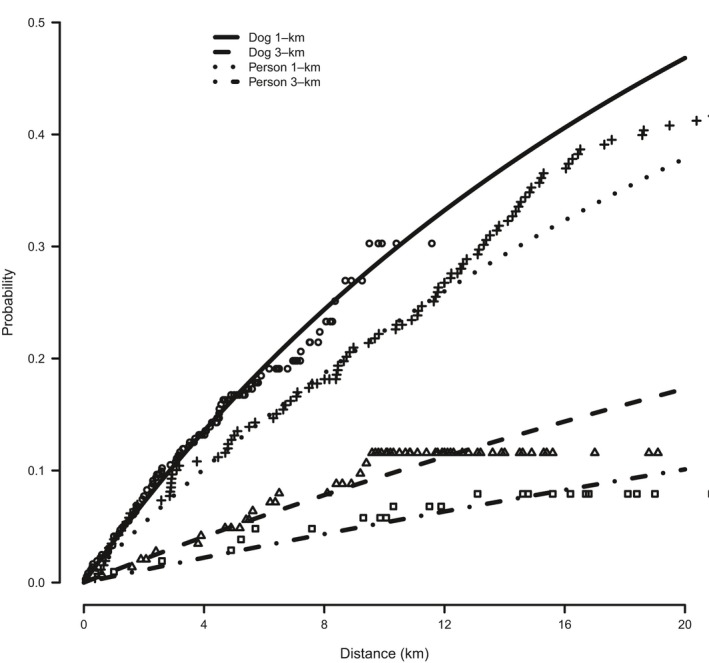
The probability of detecting a fox scat with distance searched by dog and person teams in either 1‐km or 3‐km monitoring units conditional on the total number of scats available for detection. Points are the observed proportion of scats surviving, given distance searched, calculated using the Kaplan–Meier estimator (open circles: dog team, 1‐km units; open triangles: dog team, 3‐km units; crosses: person team, 1‐km units; open squares: person team, 3‐km units)

**Figure 3:**
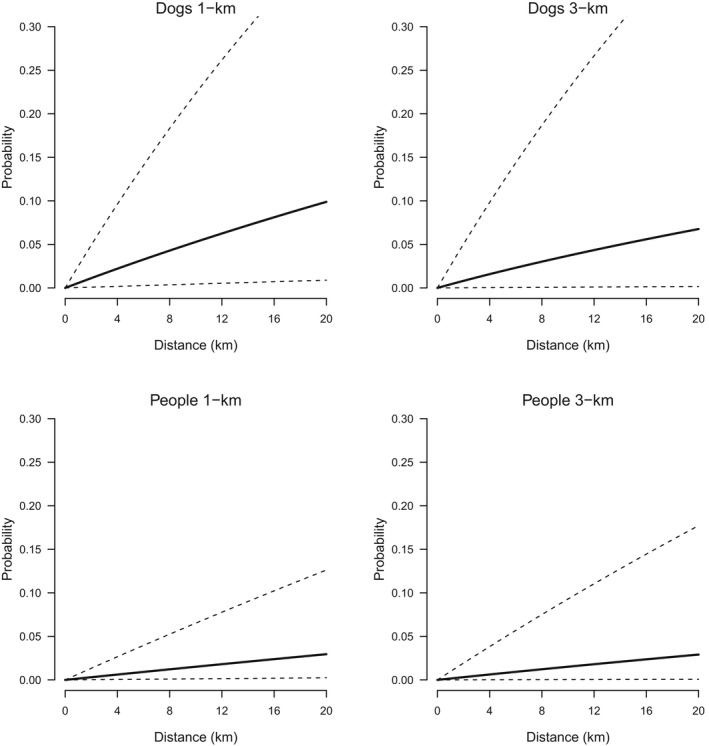
The probability of detecting a fox scat with distance searched by dog and person teams in either 1‐km or 3‐km monitoring units conditional on a single scat being available for detection (solid lines). Dashed lines are 95% credible intervals

Bayesian survival models fitted to the distance to scat detection revealed that an exponential model had a lower WAIC value than the Weibull model (difference of 6.5, *SE* = 1.41) and therefore, was a better fit to the data indicating that scat detection occurred at a relatively constant rate (Table 2). The Bayesian *p* value estimated for the exponential model was .77, indicating no strong evidence of lack‐of‐fit. The exponential model also showed a good fit to the observed data on cumulative proportion of scats detected with search distance (Figure 2). Dog teams had higher rates of scat detection than person teams, regardless of survey unit size (Table 2 and Figure 2). The resulting probabilities of detection with distance searched indicated that for dog teams, the probability of scat detection given a single scat present in the unit was 0.053 (95% CI; 0.0–0.217) and 0.037 (95% CI; 0.0–0.224) after 10 km of search effort in 1‐km and 3‐km survey units, respectively. In contrast, the corresponding detection probabilities for person teams were 0.015 (95% CI; 0.0–0.064) and 0.016 (95% CI; 0.0–0.095) after 10 km of search effort in 1‐km and 3‐km units, respectively (Table 2 and Figure 3).

**Table 2:** Parameter estimates of the detection rate for a single scat per km of search effort (log scale) for the Bayesian exponential and Weibull survival models fitted to the search distance data for each search team type (dog, person) and monitoring unit size (1‐km, 3‐km). Estimates are the mean of the posterior distribution and 95% CI is the 95% credible interval. *Shape—*Weibull shape parameter; σ—standard deviation of random effect for trial ID for each grid size; WAIC, widely applicable information criterion

Parameter	Exponential		Weibull			
	Estimate	95% CI	Estimate	95% CI	Shape	95% CI
Dog (1 km)	−5.68	−6.15, −5.24	−5.76	−6.39, −5.17	1.04	0.81, 1.28
Person (1 km)	−7.02	−7.76, −6.29	−6.99	−7.89, −6.13	0.99	0.84, 1.16
Dog (3 km)	−6.55	−7.63, −5.74	−6.53	−8.10, −5.22	1.01	0.60, 1.51
Person (3 km)	−7.46	−8.73, −6.45	−7.03	−9.11, −5.38	0.85	0.38, 1.48
σ (1 km)	1.03	0.54, 1.64	1.04	0.55, 1.66		
σ (3 km)	1.47	0.53, 2.47	1.41	0.37, 2.40		
WAIC	1952.5		1959.0			

### 3.2 Power of scat monitoring strategies to detect foxes

Track logs from unstructured and structured scat monitoring conducted at the 1 × 1 km scale between 2011 and 2015 intersected 994 monitoring units covering priority fox habitat (Figure 1a). Of these, 384 were searched by person teams, 784 were searched by dog teams, and 174 were searched by both team types. Track logs from structured scat monitoring conducted at the 3 × 3 km scale between 2011 and 2015 intersected 1,065 monitoring units covering priority fox habitat (Figure 1b). Of these, 438 were searched by person teams, 707 were searched by dog teams, and 80 were searched by both team types. Overall, person teams searched for a total of 8,044 km while dog teams searched for a total of 9,808 km. A total of 3,030 scats were collected during this period with none testing positive for fox using the mtDNA test.

**Figure 4:**
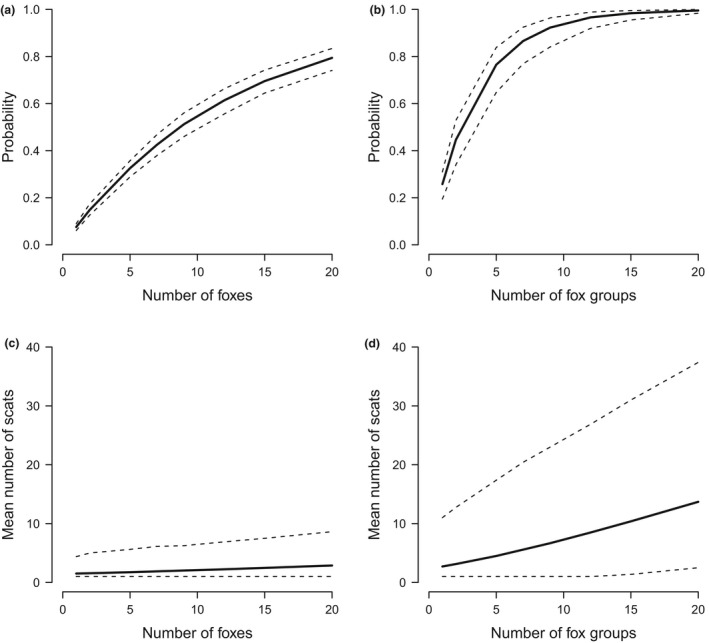
The probability that scat monitoring surveys conducted in Tasmania between 2011 and 2015 would have detected a fox versus the number of single fox or fox group home ranges estimated from 10,000 simulated fox detection surveys. (a) probability of detecting a single fox or (b) fox group; (c) mean number of fox scats detected for single foxes or (d) fox groups. Dashed lines for (a) and (b) represent the uncertainty due to variability around fox home range size and scat deposition pattern (see methods) while uncertainty around (c) and (d) are 95% credible intervals

The Monte Carlo simulation results suggest that the probability that the scat monitoring undertaken between 2011 and 2015 would have detected a single fox, given it was extant somewhere within the area of priority fox habitat, was low, at just 0.08 (range 0.06–0.09; Figure 4a). Moderate probabilities of detection (>0.50) were obtained if single foxes were present in at least 9 locations. If 20 foxes were extant independently within the area of priority fox habitat, then the probability that at least one would be detected increased to 0.80 (range 0.74–0.84; Figure 4a). In general, there was little uncertainty due to variation in scat distribution patterns within units (uniform or clumped) or variation in home range size (Figure 4a).

Probabilities of detecting a fox group were higher than detecting a single fox; the mean probability of detecting evidence of a fox, when a single group was present, was 0.26 (range 0.19–0.32) (Figure 4b). Moderate probabilities of detecting evidence of fox presence (>0.50) were obtained when fox groups were present in at least 3 locations. The probability of detecting fox presence increased to 0.80 (range 0.72–0.89) (Figure 4b) if six independent fox groups were present in the area of priority fox habitat. The mean number of fox scats detected was also higher for fox groups compared with single foxes, averaging around 2.7 scats for a single group compared with 1.5 scats for a single fox (Figure 4c,d).

The sensitivity of the estimated fox detection probability to variation in key model parameters indicated that the probability of detection was most sensitive to variation in the scat degradation rate, which explained 28% of the variance in detection probability (Appendix S4). The next highest sensitivity was due to variation in the scat detection rates for both dogs and people on 3‐km monitoring units, which explained 18% and 17% of the variance in detection, respectively, followed by the proportion of scats on linear features, which explained 5% of the variance in detection probability. The other five parameters collectively only explained 4.5% of the variance (Appendix S4).

## 4 DISCUSSION

### 4.1 Fox scat detection trials

To our knowledge, this is the first study to assess the probability of detection of individual scats by either scat‐detector dogs or person searchers under operational conditions. By operational conditions, we mean that detection trials were undertaken during a spatially and temporally extensive monitoring program (~25,000 km^2^ over 8 years) designed to detect the presence of an invasive species. As might be expected, probabilities of detecting single scats placed on linear features within a survey unit were low, at <6% after 10 km of search effort. Dog search teams had higher probabilities of detecting a scat within both 1‐km and 3‐km units compared to person search teams. However, the difference was not as large as might be expected. This is because the detection rates estimated here implicitly include the probability of scat encounter, which is equivalent to the proportion of the potential search area that was actually searched. We elaborate more on this point below.

The extensive area that could be subject to a scat search within an individual survey unit was alleviated somewhat by limiting the search to areas of “linear” habitat features. However, there were still usually many more linear features within a unit than could reasonably be searched within a day by a single search team. Consequently, the survey team selected a subset of these to search. This meant that some features in a survey unit were never searched and hence, the probability of detecting any scats on those features was effectively zero. Naturally, there can be high variation in feature “richness” between survey units which in turn would affect scat detection. However, this variation was adequately sampled within the field trials and was similar to the other factors impacting on scat detection rates: variation between searcher ability and experience (both people and dogs); variation between days due to weather conditions (temperature and humidity for dog detection); variation between seasons and substrate affecting scat degradation (detailed in Brown et al., 2014).

The probabilities of scat detection by scat‐detector dogs (and handlers) in the trials reported here are lower than have been reported in other studies (e.g., Leigh & Dominick, 2015; Long et al., 2007; Oliveira et al., 2012; Reed et al., 2011; Vynne et al., 2011). For example, Leigh and Dominick (2015) reported detection probabilities of at least 0.83 for dog detections of spotted‐tail quoll (*Dasyurus maculatus*) scats on transects in mixed vegetation types, and Reed et al. (2011) reported detection probabilities of 0.68 and 0.77 for a similar transect‐based study of multiple species of carnivore, while Fukuhara et al. (2010) reported detection probabilities of 0.92 for Indian mongoose. The main disparity between previous studies and the present study is that previous studies used scats placed on defined transects that were then subject to a search by the dog and handler. This design ensured that almost all trial scats had a very high chance of being encountered by the dog. In contrast, the trials undertaken as part of structured monitoring in the present study sought to replicate operational monitoring conditions on large survey units where scat encounter rates were likely to be low. This means our estimates of individual scat detection probabilities are likely to be more realistic for actual operational conditions involving large areas being monitored, compared to previous studies.

### 4.2 Power of scat monitoring strategies to detect foxes

Using estimates of individual scat detection probabilities and other information on fox defecation and scat degradation rates in Tasmania, we could derive realistic probabilities of detecting a fox by scat monitoring using Monte Carlo techniques. The estimates obtained on the probabilities of fox detection are relatively precise, which was mostly a reflection of having good prior estimates of some of the fundamental parameters of the simulation algorithm, such as scat degradation rates in Tasmania, scat detection rates by dog and people searches (this study) and diagnostic test sensitivity. Sensitivity analysis revealed that our estimates of fox scat monitoring power were most sensitive to variation in the scat degradation rate, followed by scat detection probabilities in 3‐km survey units. Variation due to some of the more uncertain parameters in the model such as fox home range size and scat deposition patterns had relatively minor impact on our estimates (Appendix S4). Therefore, as the parameters contributing the highest variance in model output were those where we had good estimates based on previously collected data, we were confident that our simulation algorithm adequately captured the important variability influencing the power of scat monitoring.

Using our algorithm, we examined the likelihood that scat monitoring effort undertaken from 2011 to 2015 would have detected fox populations (single foxes or family groups) of various sizes. Results suggest that, despite the high level of monitoring effort undertaken, probabilities of detecting an individual fox or fox group were low, with a probability of detecting a single fox averaging 0.08 and the probability of detecting a single fox group averaging 0.26. High probability of detection (>0.80) of fox evidence at a single location (either of a single fox or a fox group) was not obtained until at least 20 individuals, or six family groups were independently located within the area of priority fox habitat.

Whether this level of power to detect foxes using scat monitoring would be considered adequate, requires further investigation. On the one hand, a one in five chance of failing to detect up to six fox family (i.e., breeding) groups would seem to represent a significant risk for the state of Tasmania that may be unacceptable. To examine the risk more critically, it would be necessary to consider the cost of monitoring and management and the likelihood of effecting eradication of small populations of foxes versus the consequences of failing to detect and eradicate a fox incursion. Similar studies have examined the issues around determining the optimal amount of resources to allocate to monitoring and management for managing pest incursions or threatened species (Chades et al., 2008; McCarthy et al., 2010; Rout, Moore, Possingham, & McCarthy, 2011). One scenario suggests that the optimal approach may be to wait until an incursion reaches a certain size before attempting management, as the knowledge gained in the initial stages of an incursion would allow more efficient management to occur (Baxter & Possingham, 2011). However, it is generally considered that early intervention is the most cost‐effective approach to reduce the impacts from pest incursions (Bogich, Liebhold, & Shea, 2008; Rout, Kirkwood, et al., 2014).

To facilitate early detection of a fox incursion, improvements to the scat monitoring program could be considered to increase monitoring power. Although a large amount of monitoring effort was undertaken in Tasmania, the distribution of effort was not random and/or systematically distributed across all identified fox habitat, with many large areas of fox habitat remaining unmonitored (Figure 1). Here, analysis of the optimal allocation of monitoring effort could be undertaken, to determine whether it would be better to survey each monitoring unit more intensively, increase the number of units surveyed to obtain better coverage or stratify the survey effort to increase coverage toward areas more likely to be subject to an incursion (Garrard et al., 2008; Moore et al., 2011; Rout et al., 2011).

Additionally, consideration should also be given to alternative sources of monitoring information that could supplement scat monitoring. For example, passively acquired monitoring sources of fox presence produced by processes such as vehicle collisions or incidental encounters by hunters (e.g., Caley & Barry, 2014; Caley, Hosack, & Barry, 2017; Caley et al., 2015) may be a more cost‐effective and powerful option for detecting a fox incursion in Tasmania.

## CONFLICTS OF INTEREST

None declared.

## AUTHOR CONTRIBUTION

DSLR and CB conceived the ideas and designed the scat detection trials. CB, SDS, AJM, CC, ED, EM, and SQ helped collect the scat data and/or conducted the DNA testing of scats; DSLR conducted the analysis; DSLR and CB led the writing of the manuscript; All authors contributed to drafts and gave final approval for publication. We thank Peter Caley, the editor and one anonymous reviewer for helpful comments that greatly improved the manuscript. Financial support for this work was provided by the Invasive Animal Cooperative Research Centre.

## DATA ACCESSIBILITY

Data from the scat detection trials used in the survival analysis are available in supplementary materials (Appendix S1).
