# Supplementary material for: Detecting rare carnivores using scats: Implications for monitoring a fox incursion into Tasmania
Source: Ecol Evol. 2017 Dec 5;8(1):732–43. doi: 10.1002/ece3.3694 (PMC5756840; doi:10.1002/ece3.3694)
Supplement: Supplementary file 1 [file ECE3-8-732-s001.docx]

**Appendix S1 – JAGS code used for the analysis of scat detection rates**

Code to conduct the analysis of scat detection rates using JAGS is provided in the accompanying ZIP file “scat_detection_analysis.zip”, which contains two files

scat_detection_data.csv – Scat detection data with one row per scat (871 total scats). The data are organised as follows:

**team**: dog or person detector teams

**available**: Number of scat available to be detected at the site

**site**: Site ID

**grid**: 1 x 1 km or 3 x 3 km site

**group**: Integer ID for team x grid

**total.distance**: total distance searched at the site by a particular team

**distance**: distance searched for each scat (found =1) or the total distance searched (found=0)

**found**: indicator variable for a detected (found=1) or undetected (found=0) scat.

Scat_detection_analysis.R – R script to import and prepare the data, and run the JAGS models.
