# Supplementary material for: Detecting rare carnivores using scats: Implications for monitoring a fox incursion into Tasmania
Source: Ecol Evol. 2017 Dec 5;8(1):732–43. doi: 10.1002/ece3.3694 (PMC5756840; doi:10.1002/ece3.3694)
Supplement: Supplementary file 2 [file ECE3-8-732-s002.docx]

**Appendix S2 – Monte-Carlo algorithm used for simulating fox detection probability from scat searches.**

We estimated the probability that recent scat monitoring efforts in Tasmania, between 2011 and 2015, would have detected fox populations of various sizes using Monte-Carlo simulation. We systematically varied several unknown parameters related to fox biology, based on plausible values drawn from the literature to determine their effect on the probability of detection including the following:

1. Home range size of single foxes (120 ha, 470 ha, 1000 ha, 1900 ha) (Carter, Luck & McDonald 2012)
2. Distribution of scats deposited within the home range by a fox (random, clumped)

We then examined the probability that scat monitoring would have detected fox populations of different sizes, varying from 1 to 20 single foxes or fox family groups. For the purposes of this study a fox ‘group’ was defined as a family group consisting of an adult male/female pair and 4 cubs. The number of cubs is consistent with the mean litter size for adult females in Australia (Saunders *et al.* 1995). The home range size of a family group was also defined as 1.5 times the size of a single fox range size, varied as per (1) above. For each combination of fox population size, home range size and scat distribution, 10,000 replicated scat detection surveys were simulated, with each simulated survey varying the locations of foxes, the expected number of fox scats per home range (varying scat deposition and degradation), and the scat detection probability, given actual search distances (estimated from the detection trials above). The algorithm used to generate scat deposition and detection, given fox population size and (1) and (2) above, is given by the following pseudo-code.

*Pseudo-code used for the Monte-Carlo simulation algorithm*

*State-space*: Map of priority fox habitat divided into all potential 1 x 1 km monitoring units (Fig 1a)

For each iteration of the algorithm do:

1. Randomly select a 1-km monitoring unit and place a fox or fox group home range (a circle with area equal to home range size) centered on the midpoint of the cell. Determine how many monitoring units are overlapped by the fox home range.
2. Randomly generate scats (*N*) for that fox calculated as the ratio of the scat production rate and scat degradation rate (Table S1 and Appendix S2). Multiply this by the proportion of scats expected to be located on linear features (0.056).
3. Place the *N* fox scats in the monitoring units overlapped by the fox home range according to distribution type, either
   1. Random: random allocation of scats to units overlapped by fox home range
   2. Clumped: Aggregated distribution where scats are more likely to be located in the unit containing the home range centre than units on the periphery of the range. The probability *P* of a unit containing scats was given by an exponential distribution

$$P=\frac{1}{\sigma} e^{\left( - \frac{D}{\sigma} \right)}$$

   3. Where *D* is the distance of the unit from the home range centre and $\sigma$ indexes home range size.
4. Determine which monitoring units were subject to scat monitoring (i.e. actual sampled 1 × 1 or 3 × 3 km units). For sampled 3 × 3 km units, the number of fox scats in the unit was calculated by aggregating the scats from the nine 1 × 1 km units that corresponded to the location of the sampled 3 × 3 km unit.
5. If a sampled survey unit coincides with a unit occupied by a fox, then scats in the unit are detected with probability estimated from the scat detection trials. Given the actual distance searched for that unit, and the estimate of the (log) detection rate for a single scat, the corresponding probability of detecting a single scat ($p_{s}$) was given by

$$p_{s}=1-e^{- e^{r}D}$$

Where *D* was the distance searched and *r* was the (log) detection rate for a single scat, which was drawn from a log normal distribution with mean and standard deviation as given in Table S1. If *N* scats were available to be detected in the unit, then the number of scats actually detected in the unit $S_{d}$ was a binomial random variable, $S_{d} \sim Bin\left( p_{s}, N \right)$

1. Each detected ‘fox scat’ was then subject to a simulated mtDNA test which assigned the scat as belonging to a fox with a designated probability (test sensitivity). The mtDNA test was estimated to have a sensitivity (i.e. probability of test positive | fox) of 0.85 (95% CI, 0.78-0.90) (Ramsey *et al.* 2015).
2. If at least one of the detected scats evaluated as test positive, then the outcome for that iteration was recorded as 1, else 0.

End iteration

The above algorithm was simulated multiple times (*Nsim*), drawing random samples from distributions of model parameters (Table S1) at each iteration to incorporate their uncertainty in the estimated detection outcome. The probability of fox detection for this set of simulations was then calculated as the mean of the (0/1) outcomes over the *Nsim* simulations.

**Table S1**: Distributions of parameters used in the Monte-Carlo algorithm for simulating fox detection probabilities using scat monitoring, based on estimates from previous studies.

| Parameter | Distribution |
| --- | --- |
| Fox scat production rate (scats/day) | *Poisson*(8) |
| Fox scat degradation rate (day^-1^) | *LN*(-3.18, 0.32) |
| Single scat detection rate (dogs 1-km units) (km^-1^) | *LN*(-5.69, 1.03) |
| Single scat detection rate (dogs 3-km units) (km^-1^) | *LN*(-7.02, 1.47) |
| Single scat detection rate (people 1-km units ) (km^-1^) | *LN*(-6.55, 1.03) |
| Single scat detection rate (people 3-km units) (km^-1^) | *LN*(-7.46, 1.47) |
| Sensitivity of mtDNA test (probability of test positive \| fox) | *Beta*(170, 30) |
