# Supplementary material for: Detecting rare carnivores using scats: Implications for monitoring a fox incursion into Tasmania
Source: Ecol Evol. 2017 Dec 5;8(1):732–43. doi: 10.1002/ece3.3694 (PMC5756840; doi:10.1002/ece3.3694)
Supplement: Supplementary file 3 [file ECE3-8-732-s003.docx]

**Appendix S3 - Expected number of fox scats available for detection in monitoring units**

We used the methods and algorithm outlined in Brown, Ramsey and Gaffney (2014), including the estimates of fox scat degradation rates in Tasmania. For more details, the reader is referred to Brown, Ramsey and Gaffney (2014). Given that a fox is resident in an area (i.e. home range) for a period of time *t*, the expected number of scats that would be available for detection is given by

$S(t)=\frac{a}{b}+e^{-bt} \left(-\frac{a}{b}\right)$, Equation S1

Where *a* is the scat production rate/day and *b* is the scat degradation rate/day. As residence time *t* increases, the exponential component approaches zero and scat abundance reaches an equilibrium given by $a/b$. We used a Poisson distribution for the scat production rate (*a*) and log normal distribution for the (log) degradation rate (*b*) with parameters values given in Table S1. We note that only the proportion of scats deposited on linear features are actually at-risk of detection. This proportion was estimated by Webbon, Baker & Harris (2004) to be 5.6% and hence, we multiply the resulting estimate of *S*(*t*) by 0.056 to give an estimate of the abundance of scats available for detection on linear features within the home range of a single fox. The abundance of scats within the home range of a fox group was calculated similarly by multiplying the estimate for a single fox by the group size (6). Using equation S1, and parameters values in Table S1 we can estimate the average number of monitoring cells containing fox scats, for different deposition patterns (random, clumped) and home range sizes (Table S2).

**Table S2**. The expected number of monitoring cells that would contain fox scats for either 1-km or 3-km monitoring units occupied by a single fox for varying home range size and scat deposition pattern (random, clumped).

|  |  | Home range size (ha) | | | |
| --- | --- | --- | --- | --- | --- |
| Unit size | Deposition | 120 | 470 | 1000 | 1900 |
| 1-km | Random | 1.0 | 6.5 | 8.4 | 10.1 |
|  | Clumped | 1.0 | 4.7 | 7.3 | 8.0 |
| 3-km | Random | 1.0 | 1.0 | 1.0 | 4.4 |
|  | Clumped | 1.0 | 1.0 | 1.0 | 2.6 |

**References**

Brown, W.E., Ramsey, D.S.L. & Gaffney, R. (2014) Degradation and detection of fox (Vulpes vulpes) scats in Tasmania: evidence from field trials. *Wildlife Research*, **41**, 681–690.

Carter, A., Luck, G.W. & McDonald, S.P. (2012) Ecology of the red fox (*Vulpes vulpes*) in an agricultural landscape. 2. Home range and movements. *Australian Mammalogy*, **34**, 175–187.

Ramsey, D.S.L., MacDonald, A.J., Quasim, S., Barclay, C. & Sarre, S.D. (2015) An examination of the accuracy of a sequential PCR and sequencing test used to detect the incursion of an invasive species: the case of the red fox in Tasmania. *Journal of Applied Ecology*, **52**, 562–570.

Saunders, G., Kinnear, J., Braysher, M. & Coman, B. (1995) *Managing Veterbrate Pests: Foxes.* Australian Government Publishing Service, Canberra.
