# Supplementary material for: Detecting rare carnivores using scats: Implications for monitoring a fox incursion into Tasmania
Source: Ecol Evol. 2017 Dec 5;8(1):732–43. doi: 10.1002/ece3.3694 (PMC5756840; doi:10.1002/ece3.3694)
Supplement: Supplementary file 4 [file ECE3-8-732-s004.docx]

**Appendix S4: Sensitivity analysis**

We analysed the sensitivity of our model predictions of the probability of fox detection through scat monitoring to variation in the key parameters of the model. Sensitivity analysis (SA) methods can be either local or global in nature. Local SA methods are usually undertaken by varying one of the input parameters while holding other parameters constant, usually at some central value. This analysis is usually run at some central point in the space of the input parameters and hence, the volume of the region explored is nil (Saltelli et al 1999). Global SA, on the other hand, explores some finite (or infinite) region of the space of input parameters with variation in model output induced by a single input parameter examined globally by averaging over the variation of all other parameters (Saltelli et al 1999). Here we performed global SA using the Fourier amplitude sensitivity test (FAST) (Cukier *et al.* 1973). FAST is a variance based technique that computes the first order (main effects) contribution of each input parameter to the variance of the model output. These sensitivity indices (S_i_) are computed as

$S_{i}=\frac{\mathrm{var}_{X_{i}}\left[E\left(Y|X_{i}\right)\right]}{\mathrm{var}\left(Y\right)}$ Eqn. S2

Where *Y* denotes the model output, *X_i_* denotes the *i*^th^ input parameter, *E*(*Y* | *X_i_*) denotes the expectation of *Y* given *X_i_* and $\mathrm{var}_{X_{i}}$ is taken over all the possible values of *X_i_* (Saltelli et al 1999). The disadvantage of equation S2 is that evaluation for *k* input parameters requires evaluation of a *k* dimensional integral making practical application of equation 1 extremely limited. FAST resolves this issue by using a mono-dimensional Fourier decomposition along a curve exploring the *k*-dimensional search space replacing the multidimensional integrals with a one-dimensional integral evaluation. Constructing the Fourier search curve requires selecting a set of angular frequency transformations of the input parameters, usually within a predefined domain range. For a more in-depth explanation of FAST see (Saltelli Tarantola & Chan 1999; Saltelli Chan & Scott 2000).

We examined the sensitivity of the Monte-Carlo algorithm to variations in 9 key parameters using FAST. For each combination of parameters, we undertook 1000 simulated scat surveys with the target being the detection of a single randomly located fox family group. We calculated the probability of detection as the proportion of the simulated surveys that detected the fox group as an estimate of the *Y* in equation S2. The domain ranges for the parameters examined were based on the 1% and 99% percentiles of their corresponding distributions that were used in the simulations (Table S1). The parameters and domain ranges used in the sensitivity analysis are given in Table S3.

**Table S3**. Parameters used in the sensitivity analysis. Parameter values ranged between *Lower* and *Upper* values according to the FAST algorithm.

| Parameter | Description | Lower | Upper |
| --- | --- | --- | --- |
| Fox HR (ha) | Fox home range size (ha) | 120 | 1900 |
| Scat_pr | Scat production rate day^-1^ | 4 | 12 |
| Scat_dr | (log) Scat degradation rate day^-1^ | -4.75 | -1.62 |
| Dog_1km | (log) scat detection rate: dogs 1km units | -8.08 | -3.28 |
| Dog_3km | (log) scat detection rate: dogs 3km units | -9.97 | -3.14 |
| Peop_1km | (log) scat detection rate: people 1km units | -9.39 | -4.59 |
| Peop_3km | (log) scat detection rate: people 3km units | -10.9 | -4.08 |
| pLF | Proportion of scats on linear features | 0.02 | 0.1 |
| TSens | mtDNA test sensitivity | 0.7 | 0.9 |

**Results**

The sensitivity of fox detection probability to variation in each of 9 key model parameters calculated using the FAST procedure are given in Figure S1. Collectively, the first-order effects of these 9 parameters explained 72% of the variance in predicted detection probability. Hence, the unexplained portion of the variance in detection probability is due to either higher-order (interaction) effects that were not examined here or model stochasticity. The probability of detection was most sensitive to variation in the scat degradation rate (Scat_dr), which explained 28% of the variance in detection probability. The next highest sensitivity was due to variation in the scat detection rates for both dogs and people on 3-km monitoring units, which explained 18% and 17% of the variance in detection, respectively, followed by the proportion of scats on linear features, which explained 5% of the variance in detection probability. The other 5 parameters collectively only explained 4.5% of the variance.


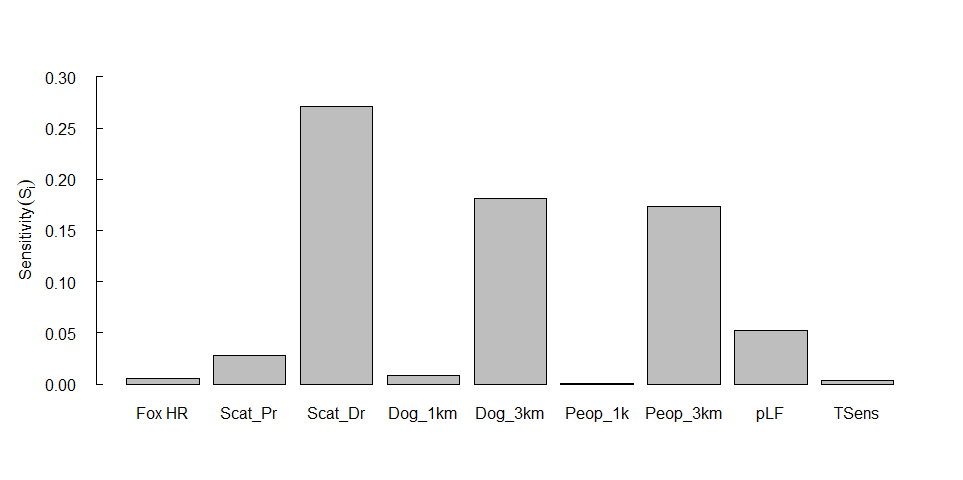


**Figure S1**. Sensitivity (S_i_ - proportional contribution to variance of model output) of the probability that scat monitoring would have detected a single fox family group to variation in each of 9 key model parameters (Table S3) calculated using the Fourier amplitude sensitivity test (FAST).

**References**

Cukier, R. I., Fortuin, C. M., Shuler, K. E., Petschek, A. G. & J.H., S. (1973) Study of the sensitivity of coupled reaction systems to uncertainties in rate coefficients. I. Theory. *Journal of Chemical Physics,* **59,** 3873-3878.

Saltelli, A., Chan, K. & Scott, E. M. (2000) Sensitivity Analysis. John Wiley & Sons, Chichester, England.

Saltelli, A., Tarantola, S. & Chan, K. (1999) A quantitative model-independent method for global sensitivity analysis of model output. *Technometrics,* **41,** 39-56.
